# Verifying the physiological significance of the preference for low facial temperature in humans

**DOI:** 10.1016/j.crphys.2025.100176

**Published:** 2025-12-13

**Authors:** Mayumi Matsuda-Nakamura, Satoshi Wada, Kei Nagashima

**Affiliations:** a Body Temperature and Fluid Laboratory, Faculty of Human Sciences, Waseda University, 2-579-15 Mikajima, Tokorozawa, Saitama, 359-1192 Japan; b Kawasaki City College of Nursing, 4-30-1 Ogura, Saiwai-ku, Kawasaki, Kanagawa, 212-0054 Japan

**Keywords:** Selective brain cooling, Tympanic temperature, Oesophageal temperature, Behavioural thermoregulation, Thermal pleasantness

## Abstract

**Objectives:**

Regional differences in thermal pleasantness have been reported; local cooling of the head induces pleasantness during mild heat exposure. However, the physiological significance of these effects remains unclear. This study tested the hypothesis that facial or neck cooling could selectively decrease brain temperature (selective brain cooling; SBC).

**Methods:**

Eight male volunteers participated in normothermic and hyperthermic protocols, comprising a 10-min baseline, 15-min cooling, and 15-min post-cooling period. During cooling, facial fanning (FAN_face_), forehead conductive cooling (COND_head_), neck conductive cooling (COND_neck_), or control without cooling (NO-C) was performed. In the hyperthermic protocol, the participants performed cycling exercises at 60 % heart rate reserve until oesophageal temperature (T_es_) rose by 1 °C. The difference between tympanic temperature (T_ty_) and T_es_ was used as an index of brain cooling.

**Results:**

Under normothermic conditions, no significant difference was observed between T_ty_ and T_es_ in any trial. Under hyperthermic conditions, T_ty_ was significantly lower than T_es_ after facial fanning, with the largest difference 10 min after the end of facial fanning (T_ty_ = 37.6 ± 0.3 °C; T_es_ = 37.7 ± 0.3 °C; *P* = 0.001, Cohen's *d* = 2.021); the brain-cooling index (T_es_–T_ty_) was 0.08 ± 0.04 °C. No significant differences occurred in the NO-C, COND_head_, or COND_neck_ trials. These results suggest that facial fanning should contribute slightly to brain cooling in hyperthermic individuals.

**Conclusion:**

A preference for low facial temperature should help cool the brain. However, this brain cooling effect should be minimal compared with SBC in animals possessing carotid rete. SBC in humans could not be validated by the results in this study.

## Highlights

• Tympanic temperature was not affected by skin temperature in normothermic condition.
• Facial fanning slightly lowered tympanic temperature below oesophageal temperature.
• A preference for low facial temperature should help to cool the brain.
• The brain-cooling effect of facial fanning should be minimal.
• Selective brain cooling in humans could not be validated.

## 1 Introduction

Humans regulate body temperature by autonomic and behavioural processes, through which skin vasodilation, sweating, taking on/off clothing, and air conditioning are involved. Cabanac suggested that in humans, conscious thermal perception of the whole body plays an important role in behavioural processes (Cabanac, 1971). Mower showed that conscious thermal perception can be categorised into two components: thermal sensation and pleasantness (discriminative and hedonic components, respectively) (Mower, 1976). Moreover, thermal pleasantness is thought to be a drive to select and search for a thermally better environment, i.e., the behavioural process of thermoregulation (Cabanac, 1971).

Changes in thermal pleasantness are generally experienced through changes in the ambient thermal environment surrounding the whole body. However, thermal stimuli for local skin areas also change thermal pleasantness. Regarding local thermal pleasantness, regional differences across body surfaces have been reported. In a hot environment, cooling of the face induces greater pleasantness than that of other skin regions; in contrast, warming the face elicits greater unpleasantness (Cotter and Taylor, 2005; Nakamura et al., 2008, 2013). We speculate that regional differences in thermal pleasantness may have physiological advantages. Behavioural thermoregulation based on the preference for low facial temperature—such as facial fanning—enhances heat dissipation from the head region. Such behavioural thermoregulation in hyperthermic individuals may help prevent heat-induced brain damage.

The brain is particularly susceptible to heat (Burger and Fuhrman, 1964). Some mammals and birds possess selective brain-cooling (SBC) mechanisms (Baker, 1979; Jessen, 2001). SBC is defined as the “lowering of brain temperature, either locally or as a whole, below the aortic (arterial blood) temperature” (IUPS Thermal Commission, 2001). Artiodactyls and felids have a carotid rete at the base of the skull, which facilitates countercurrent heat exchange and works for SBC (Jessen, 2001). However, humans do not have organs that specialise in SBC.

Despite the lack of specific anatomical organs for SBC in humans, its presence in humans remains debated (Cabanac, 1993; Matsuda-Nakamura and Nagashima, 2014; Nybo and Secher, 2011; White et al., 2011). The emissary and ophthalmic veins, upper airway, high rate of perspiration in the forehead region, and cerebrospinal fluid circulation have been suggested as components of the human SBC system (Cabanac and Brinnel, 1985; Hirashita et al., 1992; Hirata et al., 1978; Irmak et al., 2004; Mariak et al., 1999; Matsuda-Nakamura and Nagashima, 2014; Nagasaka et al., 1990; White and Cabanac, 1995). Cabanac and Caputa demonstrated that tympanic temperature (T_ty_), an index of brain temperature, was lower than oesophageal temperature (T_es_) during facial fanning in individuals with hyperthermia, suggesting the presence of SBC in humans. (Cabanac and Caputa, 1979a, 1979b). However, Nybo et al. concluded that humans do not possess the SBC system based on the results that the blood temperature difference between those running into and from the brain did not change with and without facial fanning (Nybo et al., 2002). These differences in the results might be partly attributable to methodological factors. In the study by Cabanac and Caputa, facial fanning was conducted for approximately 40 min at an ambient temperature of 10 °C (Cabanac and Caputa, 1979a), whereas in the study by Nybo et al., it was performed for 5 min at 20 °C. The 5-min duration of facial fanning might have been too short to elicit a brain-cooling effect.

Conductive cooling of the forehead or neck and facial fanning are often performed when a feeling of hotness comes with heat exposure. During heat exposure, conductive forehead cooling induces a greater reduction in hot sensation, thermal unpleasantness, and sweating than cooling of other skin regions (Cotter and Taylor, 2005; Crawshaw et al., 1975). Conductive neck cooling improves athletic performance in hot environments (Tyler and Sunderland, 2011; Tyler et al., 2010). These phenomena may be related to SBC. However, there have been no studies on SBC in humans using conductive forehead or neck cooling. In addition to facial fanning, assessing the effects of conductive cooling of the forehead and neck on SBC would be useful for identifying more effective local cooling strategies applicable to various situations such as physical work under heat stress or during exercise.

Brain temperature cannot be easily measured in healthy humans. Therefore, it has often been estimated using T_ty_. However, the use of T_ty_ as an index of brain temperature remains controversial (Mariak et al., 2003; Simon, 2007). It has been reported that T_ty_ is affected by facial skin temperature during facial fanning and does not reflect brain temperature (Nybo et al., 2002; Shiraki et al., 1988). In contrast, other studies have reported that T_ty_ is not affected by skin temperature and reflects core body temperature (Brinnel and Cabanac, 1989; Sato et al., 1996). These inconsistent findings might arise from differences in measurement methods or experimental conditions. Therefore, in each experiment, T_ty_ should be confirmed not to be affected by skin temperature when evaluating the effect of brain cooling using T_ty_.

In humans, SBC has been suggested to occur under hyperthermic conditions. When a decrease in T_ty_ is observed during facial or neck cooling in hyperthermic individuals, whether this decrease reflects a reduction in brain temperature or merely a decrease in local skin temperature near the tympanic membrane is unclear. Therefore, evaluation under hyperthermic conditions alone is insufficient. Confirming, under normothermic conditions, that T_ty_ is not influenced by skin temperature before evaluating SBC under hyperthermic conditions is necessary. However, to date, no such studies have been reported.

The present study aimed to investigate whether facial or neck cooling could induce SBC in hyperthermic humans. T_ty_ was measured as an index of brain temperature. T_es_ was measured as an index of the aortic temperature. The difference between T_ty_ and T_es_ was estimated to evaluate the effects of brain cooling. Local cooling was performed in three ways: facial fanning, conductive forehead cooling, and conductive neck cooling. To confirm the effect of skin temperature on T_ty_, local cooling trials were performed not only under hyperthermic conditions but also under normothermic conditions.

## 2 Methods

### 2.1 Participants

A total of eight male volunteers (age, 23.8 ± 3.7 years; body weight, 69.1 ± 7.1 kg; and height, 173.4 ± 4.8 cm [mean ± standard deviation (SD)]) participated in the present study. All participants were non-smokers without a clinical history of cardiovascular, metabolic, or respiratory diseases. The experimental protocol was approved by the Ethics Committee of Human Research, Waseda University (Approval Number: 2012-072), and conducted in accordance with the principles of the Declaration of Helsinki. The participants were informed about the purpose of the study and the experimental procedures involved; written informed consent was obtained from all participants before the study.

### 2.2 Experimental procedures

Four different local cooling experiments were conducted for each participant on four separate days: 1) facial fanning (FAN_face_), 2) conductive forehead cooling (COND_head_), 3) conductive neck cooling (COND_neck_), and 4) a controlled trial without local cooling (NO-C). The experiments were conducted from October to September of the following year, covering autumn to autumn. Each trial was separated by at least one week; the order was randomised. Each participant completed all trials within three months.

The day before the experiment, the participants were asked not to consume alcohol or caffeinated drinks, to avoid strenuous exercise, and to have dinner before 9 p.m. They were asked to have a light breakfast with 500 mL of water before 8 a.m. and to arrive at the laboratory by 9:30 a.m. on the day of the experiment. The participants rested in a climatic chamber maintained at an ambient temperature (T_a_) of 26 °C with a relative humidity of 30 % while wearing a tube-lined water-perfused suit (high-density water-perfusion suits; Med-Eng, Ottawa, Canada) that covered the entire body surface, except for the face, neck, hands, and feet. In addition, water-impermeable rainwear was worn over the suits to limit evaporative heat loss. The suits were continuously perfused with water (32 °C).

The participants rested on a recombinant cycle ergometer equipped with a backrest (Monark, Stockholm, Sweden) for 1 h while all measuring devices were applied. To estimate T_es_, a copper-constantan thermocouple in a polyvinyl chloride tube (Atom Multipurpose tube, 6 Fr; Atom Medical, Tokyo, Japan) was swallowed such that the tip reached the level of the left atrium (1/4 of the participant's height from the nostril). T_ty_ was measured using a thin copper-constantan thermocouple with a tip covered with a thin layer of absorbent cotton. The participants advanced the thermocouple into the ear canal until the tympanic membrane was reached, as previously reported (Sato et al., 1996). When the probe sensor came into contact with the tympanic membrane, the participants experienced slight sharp pain and heard a scratching noise. The ear probe was carefully positioned until the highest stable temperature was obtained, which was very close to T_es_ (Fig. 2). The ear canal was then closed with absorbent cotton, and the entire ear was covered with sound insulation earmuffs. Skin temperature was measured using copper-constantan thermocouples at eight skin sites: the forehead (T_head_), chest (T_chest_), forearm (T_arm_), hand (T_hand_), thigh (T_thigh_), lower leg (T_leg_), foot (T_foot_), and neck (T_neck_). The temperature was recorded every 10 s. Heart rates (HR) were continuously assessed by electrocardiography (Life Scope BSM-2401; NIHON KOHDEN, Tokyo, Japan) and recorded every 5 min. The blood pressure in the upper arm was measured every 5 min using a sonometric pickup (STBP-780; Colin, Komaki, Japan). Skin blood flow in the forehead and forearm was assessed using laser Doppler flowmetry (LDF_head_ and LDF_arm,_ respectively; ALF 21; Advance, Tokyo, Japan) every 1 s. The sweat rate at the forearm (SR_arm_) was measured using dew hygrometry (POS-02; Skinos Giken, Nagoya, Japan) every 1 s.

**Fig. 1:**
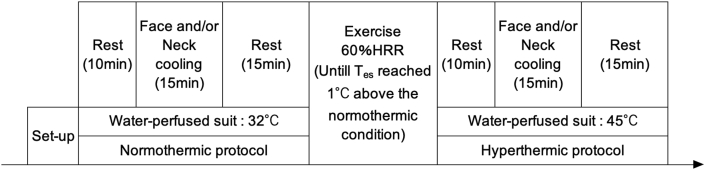
Schema of the experimental time course.

In each trial, the participants underwent local cooling under normothermic (normal body temperature; normothermic protocol) and hyperthermic (higher body temperature; hyperthermic protocol) conditions after the resting period (Fig. 1). First, they underwent 40-min normothermic protocols, while water at 32 °C continuously perfused the suits. The protocol comprised a 10-min baseline and 15-min local cooling period, followed by a 15-min period without cooling. During the cooling period, FAN_face_, COND_head_, COND_neck_, or NO-C was performed. During FAN_face_, two fans were placed at a distance of 0.5 m from each participant so that the wind came from right and left angles of 45°, respectively. In addition, the wind direction was adjusted to blow on the face and neck; rainwear insulated the rest of the body's surface. The diameter of the fan was 0.35 m; the airspeed was 2 m/s at the face. COND_head_ and COND_neck_ measurements were conducted using water-perfused vinyl tubes (7 mm in diameter and 1 mm in thickness) on a thermally conductive sheet (GP1-0.5; Kitagawa Industries, Inazawa, Japan). The plain face of the sheet was attached to the skin. One piece of sheet (10 cm long, 20 cm wide, 200 cm^2^) was used for the COND_head_, whereas three pieces of sheet (one 10 × 10 cm, 100 cm^2^; and two 10 × 5 cm sheets, 100 cm^2^) were used for the COND_neck_. The water temperature was 20 °C for both COND_head_ and COND_neck_ and was maintained at 33 °C for the rest of the period.

**Fig. 2:**
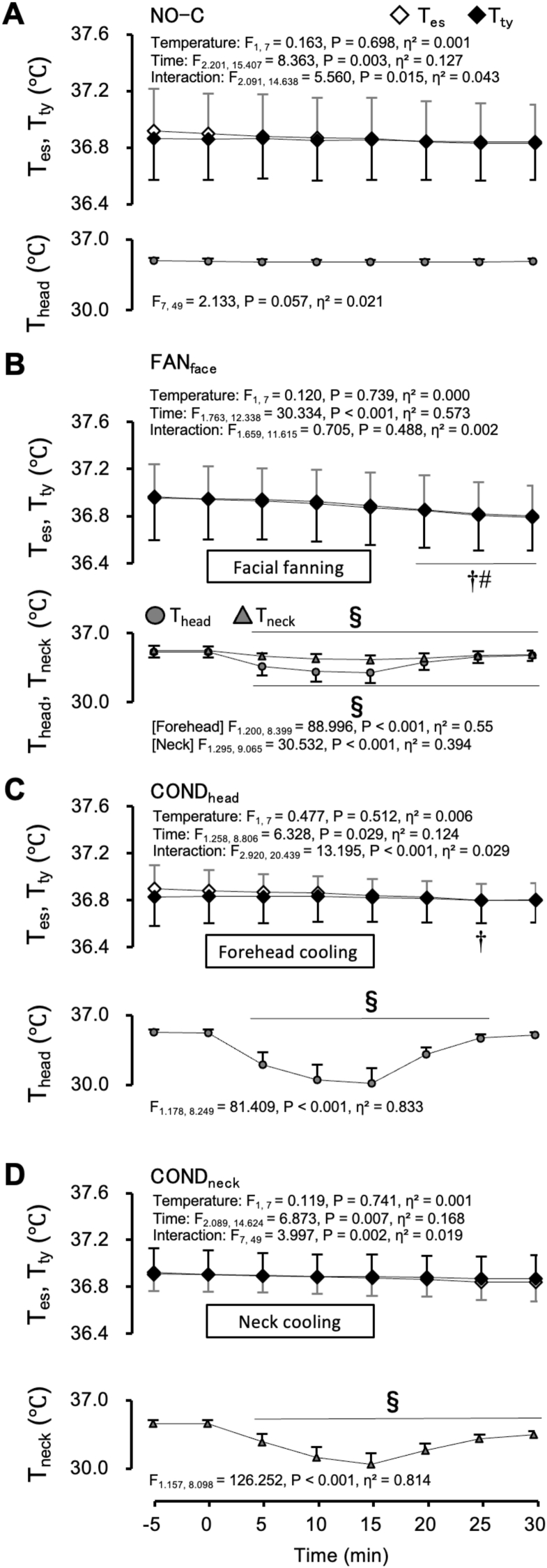
Oesophageal temperature (T_es_), tympanic temperature (T_ty_), and skin temperature of the cooling area under normothermic conditions. Values represent the mean ± SD (n = 8). Symbols indicate a significant difference (*P* < 0.05): † : vs. 0 min T_es_; # : vs. 0 min T_ty_ value; § : vs. 0 min skin temperature of the cooling area.

After the normothermic protocol, the participants drank 100 mL of water and underwent a hyperthermic protocol. The participants performed cycling exercises at a pedalling rate of 60 rpm. The heart rate was maintained at 60 % of the heart rate reserve (HRR). HRR (%) was calculated as follows: (HR during exercise − HR during rest)/(maximum HR − HR during rest) × 100. Since we did not measure the maximum heart rate of each participant, the value was estimated as (220–age of the participants) (Astrand et al., 1986). When T_es_ reached 1 °C above the baseline, the perfusion water temperature for the suit was changed to 45 °C; the participants were asked to stop pedalling. After the HR stabilised, a 15-min local cooling period followed by a 15-min period without cooling was conducted in the same manner according to the normothermic protocol.

### 2.3 Measurement of thermal perception

Local thermal sensation and pleasantness at the cooled sites, as well as whole-body thermal sensation and pleasantness, were assessed every 5 min and 2 min after the start and end of local cooling using a 10 cm visual analogue scale. In the ratings of thermal sensation and pleasantness, −10 was defined as “maximally cold” or “maximally pleasant”, 10 as “maximally hot” or “maximally unpleasant”, and 0 as “neutral”. The participants were instructed to differentiate thermal sensation from pleasantness carefully; we asked them to report current thermal perception, i.e., “how much they feel the skin surface temperature has increased or decreased (thermal sensation) and how much they like or dislike the thermal condition (thermal pleasantness)”, respectively.

### 2.4 Calculation and statistics

The weighted mean skin temperature (mean T_sk_) was 0.07 T_head_ + 0.35 T_chest_ + 0.14 T_arm_ + 0.05 T_hand_ + 0.19 T_thigh_ + 0.13 T_leg_ + 0.07 T_foot_, based on the formula by Hardy et al. (1938). The mean arterial pressure (MAP) was calculated as (systolic pressure − diastolic pressure)/3 + diastolic pressure. Cutaneous vascular conductance (CVC) at the forehead (CVC_head_) and forearm (CVC_arm_) was calculated as LDF/MAP. Changes in LDF and CVC were expressed as percentage changes from the average values at −5 to 0 min (defined as 100 %). All values, except for HR, MAP, and sensation ratings, were averaged every 5 min.

Differences between T_es_ and T_ty_ were evaluated using two-way repeated-measures analysis of variance (ANOVA). Changes in skin temperature for each cooled site were evaluated using one-way repeated-measures ANOVA. Differences among the four trials were evaluated using two-way repeated-measures ANOVA. In all repeated-measures ANOVA, we tested whether Mauchly's sphericity assumption was violated. If the result of Mauchly's test was significant and the assumption of sphericity was violated, the Greenhouse-Geisser adjustment was used for correcting sphericity by altering the degrees of freedom using a correction coefficient epsilon. A post-hoc test using the Bonferroni method was performed when a significant main effect or interaction was observed. Correlations between skin temperature and T_ty_ in individual participants under the normothermic protocol were evaluated using Pearson's correlation coefficients. The null hypothesis was rejected at *P* < 0.05. The effect sizes were calculated as eta squared (*η*^2^) for the two-way repeated measures ANOVA and Cohen's *d* values for the paired comparisons derived from Bonferroni-adjusted post hoc tests (Lakens, 2013). The effect size was interpreted conservatively as small (*η*2 = 0.01, Cohen's *d* = 0.20), medium (*η*2 = 0.06, Cohen's *d* = 0.50) or large (*η*2 = 0.14, Cohen's *d* = 0.80) (Cohen, 1988; Lakens, 2013). The statistical results are presented in the figures or tables. Data are presented as mean ± standard deviation (SD).

## 3 Results

Fig. 2 shows the T_es_, T_ty_, and skin temperature of the cooling area during the NO-C (A), FAN_face_ (B), COND_head_ (C), and COND_neck_ (D) trials of the normothermic protocol, respectively. Time zero denotes the onset of each cooling process. During the NO-C trial, T_es_, T_ty_, and T_head_ remained unchanged from 0 to 30 min (Fig. 2A). During the COND_neck_ trial, T_es_ and T_ty_ remained unchanged during the 30-min protocol (Fig. 2D). However, during the FAN_face_ trial, T_es_ and T_ty_ decreased from their values at 0 min to 20–30 min (Fig. 2B, *P* < 0.05). During the COND_head_ trial, the T_es_ decreased at 25 min from its initial value at 0 min (Fig. 2C, *P* = 0.041). During all four trials, no significant differences were observed between T_es_ and T_ty_. T_ty_ was very close to T_es_; for instance, in the FAN_face_ trial, baseline values (at 0 min) were 37.0 ± 0.3 °C for T_ty_ and 36.9 ± 0.3 °C for T_es_. Each cooling procedure reduced skin temperature of the cooling area from its value at 0 min. The cooling effect on skin temperature lasted for up to 30 min on the FAN_face_ (Fig. 2B, *P* < 0.05) and COND_neck_ trials (Fig. 2D, *P* < 0.001) and 25 min in the COND_head_ trial (Fig. 2C, *P* < 0.05).

Table 1 presents the correlation coefficients (*R*) between skin temperature and T_ty_, together with the corresponding probability values (*P*), for each participant under each cooling condition in the normothermic protocol. No significant positive correlations were observed.

**Table 1:** Correlation coefficients (*R*) between skin temperature and T_ty_ and their probability values (*P*) for each participant under the normothermic protocol.

Participants no.	FAN_face_		COND_head_		COND_neck_	
	*R*	*P*	*R*	*P*	*R*	*P*
1	−0.423	0.344	−0.664	0.104	−0.471	0.286
2	−0.476	0.280	−0.090	0.848	0.224	0.628
3	−0.239	0.605	−0.680	0.093	0.251	0.588
4	−0.290	0.528	−0.256	0.579	−0.369	0.416
5	0.164	0.726	−0.586	0.167	−0.416	0.353
6	−0.343	0.451	−0.401	0.373	0.698	0.081
7	−0.858	0.014	0.350	0.441	0.305	0.506
8	0.355	0.434	−0.705	0.077	−0.595	0.158

Mean	−0.26	0.42	−0.38	0.34	−0.05	0.38
SD	0.38	0.22	0.37	0.28	0.47	0.20

Values were calculated using the data obtained from 0 to 30 min.

**Fig. 3:**
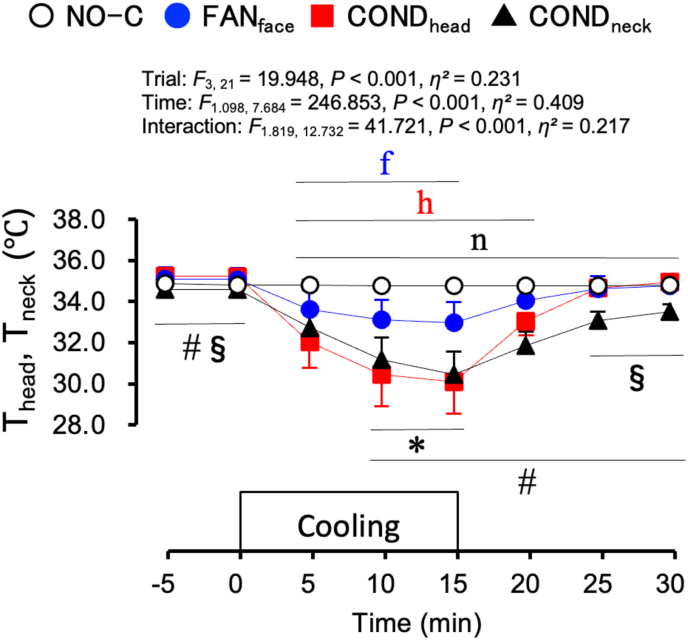
Skin temperature of the cooling area (T_head_ for the NO-C, FAN_face_, and COND_head_ trials, and T_neck_ for the COND_neck_ trial) under normothermic conditions. Values represent the mean ± SD (n = 8). Symbols indicate a significant difference (*P* < 0.05): f : NO-C vs. FAN_face_; h : NO-C vs. COND_head_; n : NO-C vs. COND_neck_; ∗ : FAN_face_ vs. COND_head_; # : FAN_face_ vs. COND_neck_; § : COND_head_ vs. COND_neck_.

Fig. 3 shows skin temperature of the cooling area (T_head_ in the NO-C, FAN_face_, and COND_head_ trials, and T_neck_ in the COND_neck_ trial) under the normothermic protocol. The skin temperature of the cooling area decreased during each local cooling period and was lower during the FAN_face_, COND_head_, and COND_neck_ trials than during the NO-C trial after the start of local cooling (*P* < 0.05). The cooling effect reached a maximum at 15 min in each cooling trial; the reduction was the greatest during the COND_head_ trial (5.1 ± 1.4 °C from the value at 0 min).

**Fig. 4:**
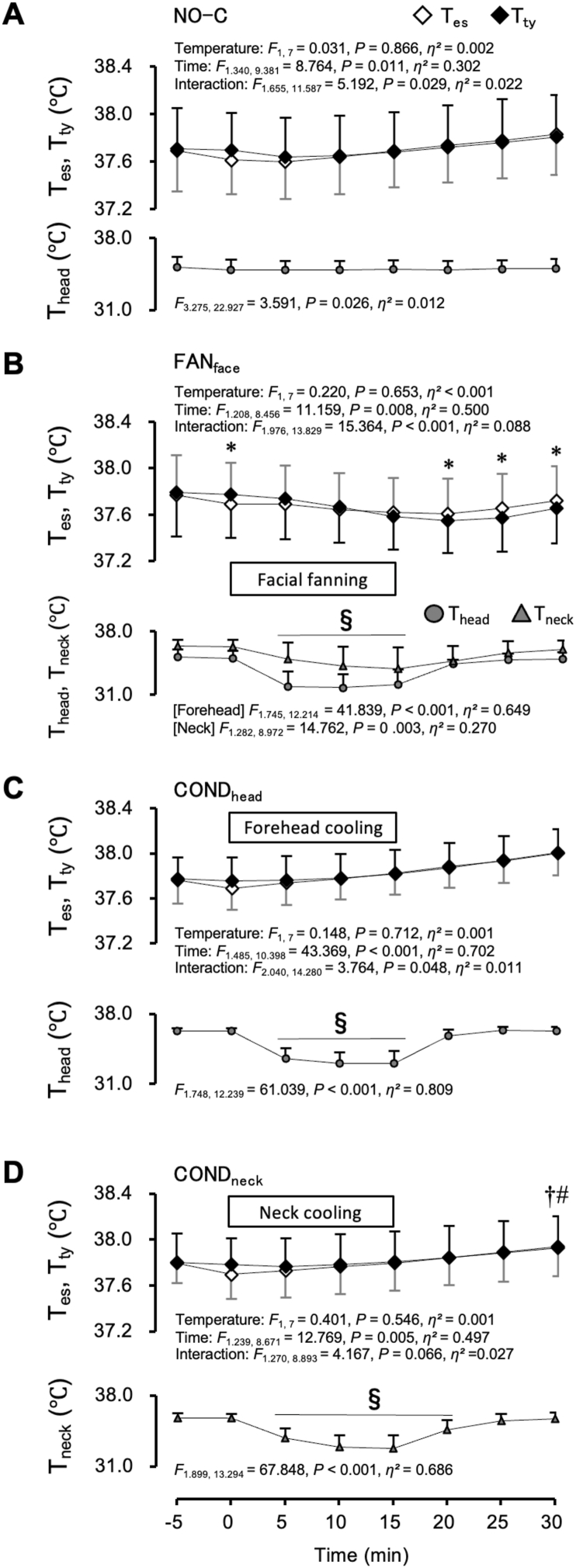
Oesophageal temperature (T_es_), tympanic temperature (T_ty_), and skin temperature of the cooling area under hyperthermic conditions. Values represent the mean ± SD (n = 8). Symbols indicate a significant difference (*P* < 0.05): ∗ : T_es_ vs. T_ty_; † : vs. 0 min T_es_; # : vs. 0 min T_ty_; § : vs. 0 min skin temperature of the cooling area.

Fig. 4 shows the T_es_, T_ty_, and skin temperature of the cooling area during the NO-C (A), FAN_face_ (B), COND_head_ (C), and COND_neck_ (D) trials under the hyperthermic protocol, respectively. Time zero denotes the onset of each cooling process. To reach T_es_ of >1.0 °C, it took 21.2 ± 3.9 min from the onset of the exercise. The workload of the exercise was 105.5 ± 22.4 W. The period required for the HR plateau was 9.4 ± 1.7 min; the mean HR plateau value was 99.1 ± 12.4 beats/min. During the four trials, T_es_ and T_ty_ were greater than the values at 0 min under the normothermic protocols shown in Fig. 2. During the NO-C trial, T_es_ and T_ty_ were 36.9 ± 0.3 °C and 36.9 ± 0.3 °C, respectively, at 0 min in the normothermic protocol; these increased by 0.7 ± 0.1 °C and 0.9 ± 0.1 °C, respectively, at 0 min in the hyperthermic protocol. Significant differences between T_es_ and T_ty_ were observed during the FAN_face_ trial (Fig. 4B). The difference was the greatest at 25 min; T_ty_ (37.6 ± 0.3 °C) was lower than T_es_ (37.7 ± 0.3 °C, *P* = 0.001, Cohen's *d* = 2.021), and the index of brain cooling (difference between T_es_ and T_ty_) was 0.08 ± 0.04 °C. Table 2 summarises individual values of T_es_ and T_ty_ at 0 and 25 min under the hyperthermic protocol. At 0 min, T_ty_ was higher than T_es_ in seven of the eight participants, whereas at 25 min, T_ty_ was lower than T_es_ in all eight participants. On average, T_es_ decreased by 0.04 °C, whereas T_ty_ decreased by 0.20 °C. Although the difference between T_es_ and T_ty_ at 25 min was only 0.08 °C, the effect size (Cohen's *d* = 2.021) indicated a very large effect. In contrast to the FAN_face_ trial, no significant differences were observed between T_es_ and T_ty_ in the other three trials (Fig. 4A–C, and D). In the COND_neck_ trial, T_es_ and T_ty_ increased at 30 min from their values at 0 min (Fig. 4D, *P* = 0.033). However, during the other three trials, T_es_ and T_ty_ did not change significantly from their values at 0 min (Fig. 4A–C). Each cooling procedure reduced skin temperature of the cooling area from its value at 0 min. A cooling effect on skin temperature was observed in the FAN_face_ and COND_head_ at 5–15 min (Fig. 4B and C, *P* < 0.05) and 5–20 min during the COND_neck_ trial (Fig. 4D, *P* < 0.05).

**Table 2:** Individual values of T_es_ and T_ty_ at 0 and 25 min under the hyperthermic protocol.

Participants no.	0m		25m		Change（25m–0m）		Difference（T_es_ - T_ty_）	
	T_es_	T_ty_	T_es_	T_ty_	ΔT_es_	ΔT_ty_	0m	25m
1	37.22	37.34	37.27	37.18	0.05	−0.16	−0.11	0.09
2	37.62	37.63	37.77	37.63	0.15	0.00	−0.01	0.14
3	37.82	37.97	37.61	37.60	−0.20	−0.37	−0.15	0.01
4	37.96	38.10	37.82	37.74	−0.15	−0.36	−0.14	0.08
5	37.63	37.68	37.66	37.54	0.02	−0.14	−0.05	0.12
6	38.36	38.45	38.24	38.16	−0.12	−0.29	−0.09	0.08
7	37.45	37.41	37.40	37.33	−0.05	−0.07	0.05	0.07
8	37.49	37.63	37.47	37.43	−0.02	−0.20	−0.14	0.04

Mean	37.69	37.77	37.65	37.57	−0.04	−0.20	−0.08	0.08
SD	0.35	0.37	0.30	0.29	0.12	0.13	0.07	0.04
Cohen's *d*							−1.156	2.021

Cohen's *d* values represent the effect sizes for the comparison between T_es_ and T_ty_.

**Fig. 5:**
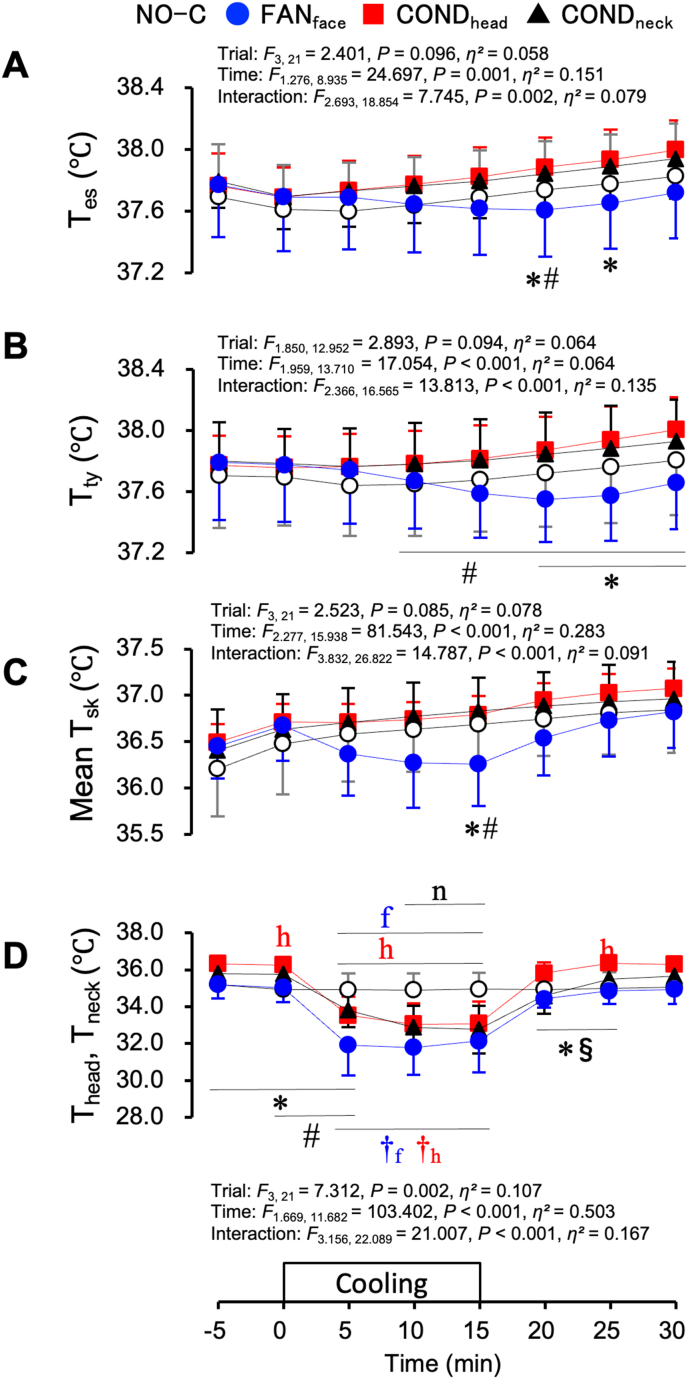
Oesophageal temperature (T_es_, A), tympanic temperature (T_ty_, B), mean skin temperature (Mean T_sk_, C), and skin temperature (D) of the cooling area (T_head_ for the NO-C, FAN_face_, and COND_head_ trials, and T_neck_ for the COND_neck_ trial) under hyperthermic conditions. Values represent the mean ± SD (n = 8). Symbols indicate a significant difference (*P* < 0.05): f : NO-C vs. FAN_face_; h : NO-C vs. COND_head_; n : NO-C vs. COND_neck_; ∗ : FAN_face_ vs. COND_head_; # : FAN_face_ vs. COND_neck_; § : COND_head_ vs. COND_neck_; †_f_ : vs. 0 min in the FAN_face_ trial; †_h_ : vs. 0 min in the COND_head_ trial.

Fig. 5 shows the T_es_ (A), T_ty_ (B), mean T_sk_ (C), and skin temperature of the cooling area (D, T_head_ during the NO-C, FAN_face_, and COND_head_ trials, and T_neck_ during the COND_neck_ trial) in the hyperthermic protocol. There were no differences in T_ty_, T_es_, or mean T_sk_ at 0 min among the four trials. After the start of local cooling, differences among the four trials were observed. T_es_ was lower during the FAN_face_ trial than during the COND_head_ trial at 20–25 min (Fig. 5A, *P* < 0.05) and lower than that during the COND_neck_ trial at 20 min (*P* = 0.025). T_ty_ was lower during the FAN_face_ trial than during the COND_head_ trial at 20–30 min (Fig. 5B, *P* < 0.01) and lower than that during the COND_neck_ trial at 10–30 min (*P* < 0.05). The mean T_sk_ was lower during the FAN_face_ trial than during the COND_head_ and COND_neck_ trials at 15 min (Fig. 5C, *P* < 0.05). The skin temperature of the cooling area was higher during the COND_head_ trial than during the NO-C trial and lower in the FAN_face_ trial than during the COND_head_ and COND_neck_ trials at 0 min (Fig. 5D, *P* < 0.05). After starting local cooling, skin temperature of the cooling area was lower during the FAN_face_, COND_head_, and COND_neck_ trials than during the NO-C trial (*P* < 0.05). The reduction in skin temperature was the greatest at 10 min during the FAN_face_ trial (3.5 ± 1.0 °C from the value at 0 min).

**Fig. 6:**
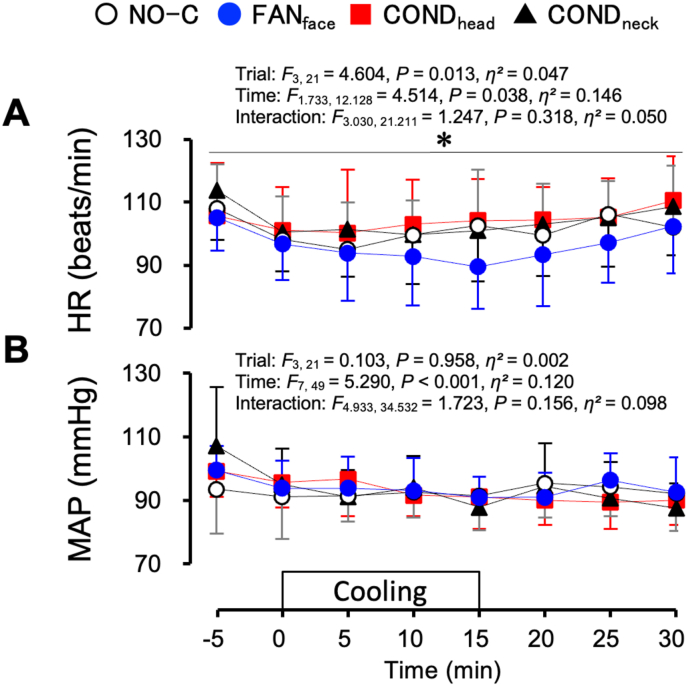
Heart rate (HR, A) and mean arterial pressure (MAP, B) under hyperthermic conditions. Values represent the mean ± SD (n = 8). Symbols indicate a significant difference (*P* < 0.05): ∗ : FAN_face_ vs. COND_head_.

Fig. 6 shows the HR (A) and MAP (B) in the hyperthermic protocol. The HR was lower throughout the FAN_face_ trial than during the COND_head_ trial (Fig. 6A, *P* = 0.037). MAP did not differ among the four trials (Fig. 6B).

**Fig. 7:**
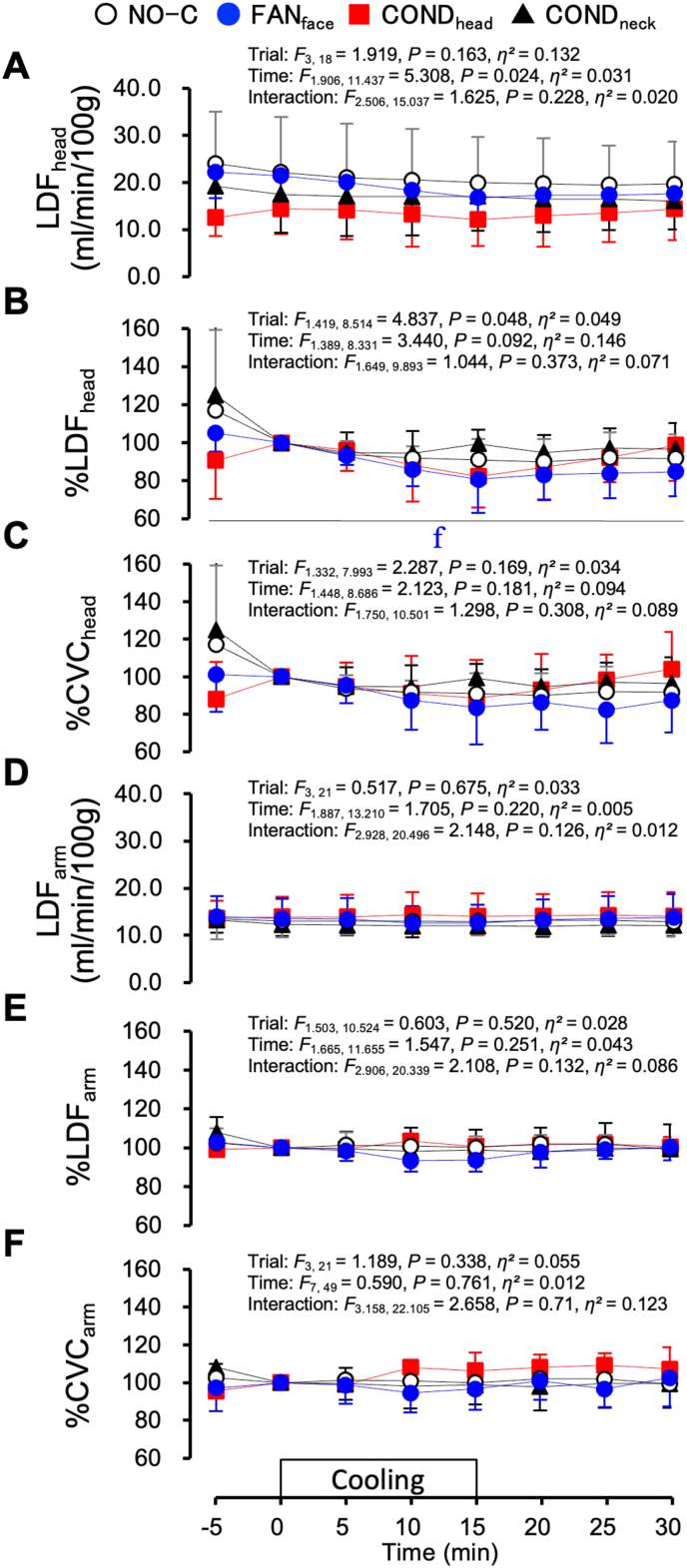
Laser-Doppler flow (LDF) at the forehead (LDF_head_, A, n = 7), percentage change in the LDF_head_ (%LDF_head_, B, n = 7), percentage change in cutaneous vascular conductance (CVC) at the forehead (%CVC_head_, C, n = 7), LDF at the forearm (LDF_arm_, D, n = 8), percentage change in the LDF_arm_ (%LDF_arm_, E, n = 8), and percentage change in CVC at the forearm (%CVC_arm_, F, n = 8) under hyperthermic conditions. One hundred percent for LDF and CVC denotes the average value from −5 to 0 min. Values represent mean ± SD. Symbols indicate a significant difference (*P* < 0.05): f : NO-C vs. FAN_face_.

Fig. 7 shows the LDF and CVC under the hyperthermic protocol. The LDF_head_ could not be measured for one participant. Hence, the mean values in the seven participants are shown in the LDF_head_ and CVC_head_ (Fig. 7A–C). Using the absolute values of the LDF collected on separate days is not appropriate because the adhesive force and position of the probe could influence the values. However, to represent the percentage changes in LDF, the absolute values of LDF are shown (Fig. 7A and D). LDF_head_ did not differ among the four trials (Fig. 7A). The percentage change in LDF_head_ (%LDF_head_) was lower throughout the FAN_face_ trial than during the NO-C trial (Fig. 7B, *P* = 0.027). The percentage change in CVC_head_ (%CVC_head_) did not differ among the four trials (Fig. 7C). The percentage changes in LDF_arm_ (%LDF_arm_) and CVC_arm_ (%CVC_arm_) were not significantly different among the four trials (Fig. 7E and F).

**Fig. 8:**
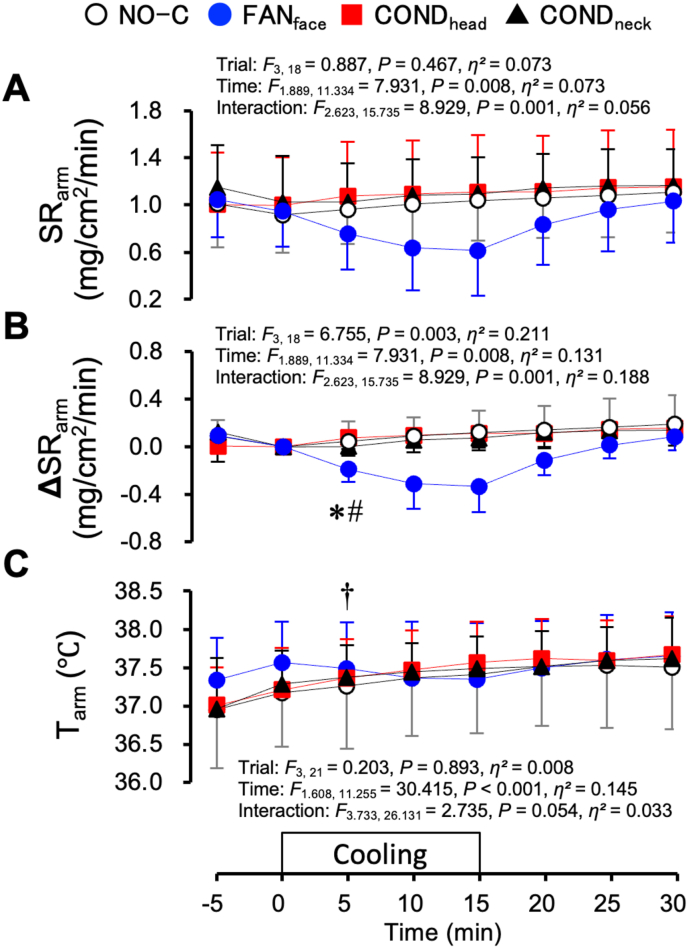
Sweat rate at the forearm (SR_arm_, A), change in sweat rate (ΔSR_arm_, B), and skin temperature of the forearm under hyperthermic conditions. Values represent the mean ± SD (n = 7). Symbols indicate a significant difference (*P* < 0.05): ∗ : FAN_face_ vs. COND_head_; # : FAN_face_ vs. COND_neck_; † : vs. 0 min (no significant differences among the four trials).

Fig. 8 shows the SR_arm_ and T_arm_ under the hyperthermic protocol. SR_arm_ could not be measured for one participant. The mean values for the seven participants are shown. SR_arm_ did not differ across the four trials (Fig. 8A). However, the change in SR_arm_ (ΔSR_arm_) was lower during the FAN_face_ trial than during the COND_head_ (*P* = 0.004) and COND_neck_ (*P* = 0.029) trials at 5 min (Fig. 8B). T_arm_ increased at 5 min from the value at 0 min without significant differences among the four trials (Fig. 8C, *P* = 0.032).

**Fig. 9:**
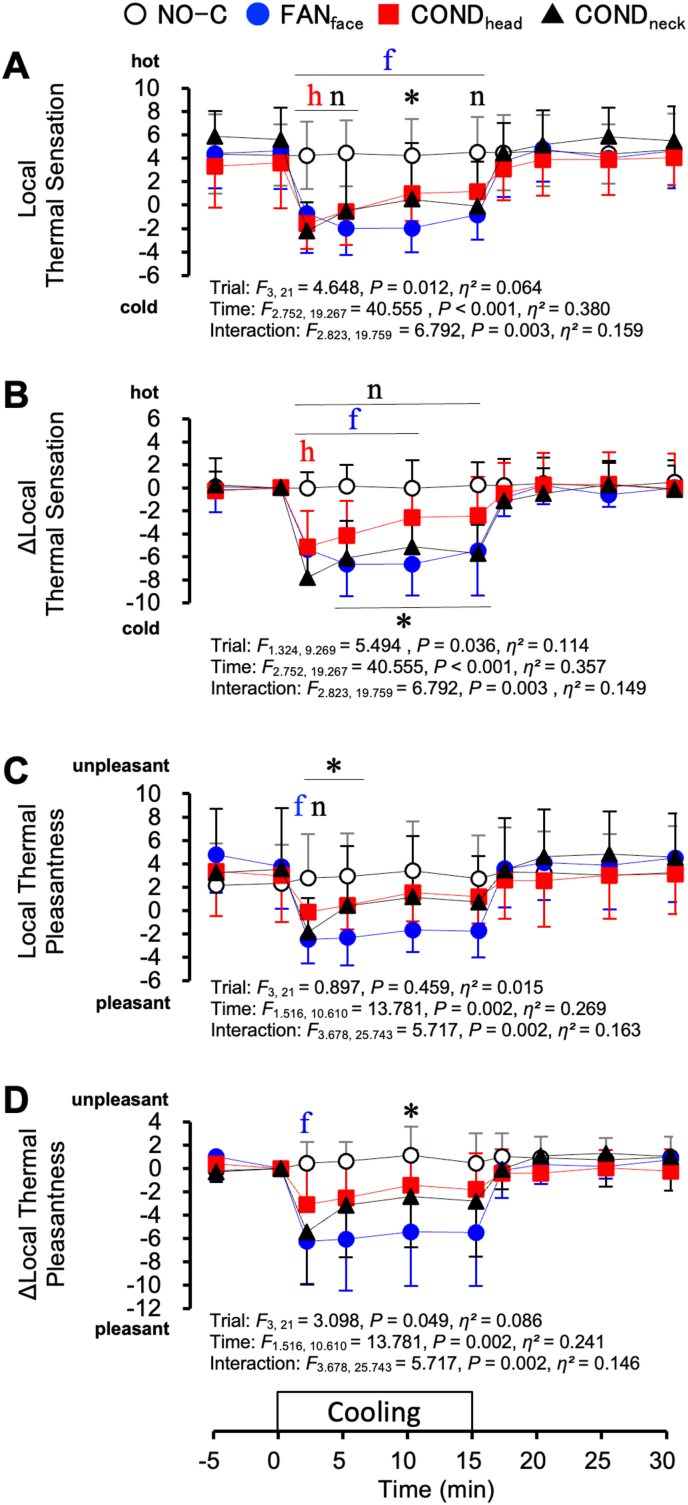
Local thermal sensation (A), change in local thermal sensation (Δlocal thermal sensation, B), local thermal pleasantness (C), and change in local thermal pleasantness (Δlocal thermal pleasantness, D) under hyperthermic conditions. Values represent mean ± SD (n = 8). Symbols indicate a significant difference (*P* < 0.05): f : NO-C vs. FAN_face_; h : NO-C vs. COND_head_; n : NO-C vs. COND_neck_; ∗ : FAN_face_ vs. COND_head_.

Fig. 9 shows the rating scores of the local thermal sensation and pleasantness for the cooling area under hyperthermic conditions. The “hot” and “unpleasant” ratings were expressed as positive values. The local thermal sensation for the cooling area was significantly colder during the FAN_face_ (2–15 min), COND_head_ (2–5 min), and COND_neck_ (2–5 min and at 15 min) trials than during the NO-C trial (all *P* < 0.05; Fig. 9A). The change in local thermal sensation (Δlocal thermal sensation) was also colder during the FAN_face_ (2–10 min, *P* < 0.05), COND_head_ (2min, *P* = 0.049), and COND_neck_ (2–15 min, *P* < 0.001) trials than during the NO-C trial (Fig. 9B). Regarding the local thermal pleasantness, a more pleasant sensation was reported during the FAN_face_ (*P* = 0.03), and COND_neck_ (*P* = 0.035) trials than during the NO-C trial at 2 min (Fig. 9C). The change in local thermal pleasantness (Δlocal thermal pleasantness) was also rated as more pleasant during the FAN_face_ trial than during the NO-C trial at 2 min (Fig. 9D, *P* = 0.022).

**Fig. 10:**
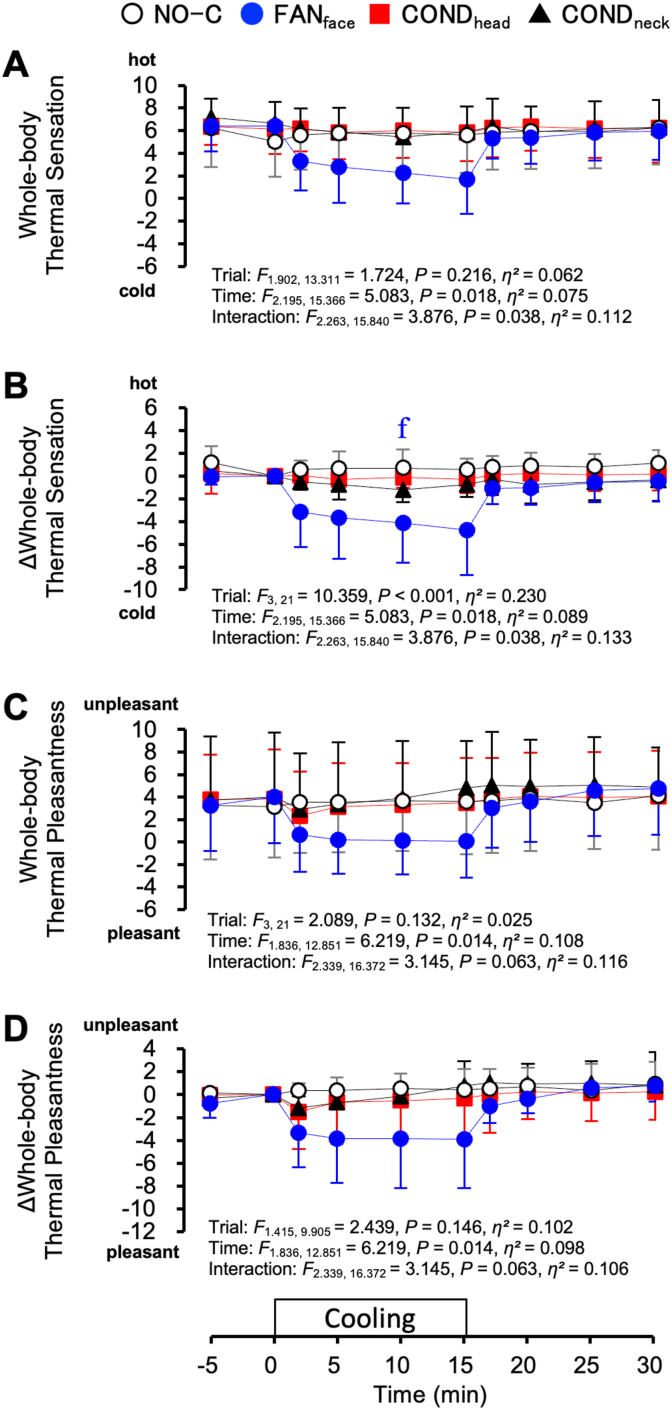
Whole-body thermal sensation (A), change in whole-body thermal sensation (Δwhole-body thermal sensation, B), whole-body thermal pleasantness (C), and change in whole-body thermal pleasantness (Δwhole-body thermal pleasantness, D) under hyperthermic conditions. Values represent the mean ± SD (n = 8). Symbols indicate a significant difference (*P* < 0.05): f : NO-C vs. FAN_face_.

Fig. 10 shows the rating scores of the whole-body thermal sensation and pleasantness under hyperthermic conditions. The whole-body thermal sensation did not differ among the four trials (Fig. 10A). However, the change in whole-body thermal sensation (Δwhole-body thermal sensation) was greater during the FAN_face_ trial than during the NO-C trial at 10 min (Fig. 10B, *P* = 0.042). Regarding whole-body thermal pleasantness and its changes (Δwhole-body thermal pleasantness), there were no statistically significant differences among the four trials (Fig. 10C and D).

## 4 Discussion

In the present study, we aimed to investigate whether face or neck cooling could induce SBC in hyperthermic humans. Three local cooling methods (FAN_face_, COND_head_, and COND_neck_) and time control without cooling (NO-C) were performed in normal and hyperthermic participants. T_ty_ and T_es_ were measured as indices of brain and aortic temperatures, respectively. The difference between T_ty_ and T_es_ was estimated to evaluate the brain cooling effect. Under the normothermic protocol, no significant difference was observed between T_ty_ and T_es_ during any of the four trials (Fig. 2A–D). Under the hyperthermic protocol, T_ty_ was slightly lower than T_es_ after facial fanning (Fig. 4B).

### 4.1 Effect of skin temperature on T_ty_

Under normothermic conditions, skin temperature of the cooling area decreased immediately after starting local cooling. However, T_ty_ did not change during the 15-min local cooling period (Fig. 2B–D). In addition, there was no difference between T_ty_ and T_es_ during the four trials (Fig. 2A–D). In addition, under normothermic conditions, no significant positive correlations were observed between skin temperature and T_ty_ in any participant (Table 1). These results suggest that T_ty_ is not affected by skin temperature and reflects the precise core body temperature during face or neck cooling under normothermic conditions.

### 4.2 Effect of facial fanning on brain cooling and human SBC

We estimated the difference between T_ty_ and T_es_ to evaluate the effects of brain cooling. Under the hyperthermic protocol, T_ty_ was lower than T_es_ after facial fanning (Fig. 4B). This result suggests that facial fanning can cool the brain of hyperthermic humans. However, the maximum index of brain cooling (difference between T_ty_ and T_es_) was only 0.08 ± 0.04 °C. Cabanac and Caputa demonstrated that T_ty_, an index of brain temperature, was >1 °C lower than T_es_ during facial fanning in hyperthermic humans (Cabanac and Caputa, 1979a). Some reasons for the difference in results between the present study and previous study may depend on the experimental methods. For instance, in this study, the airspeeds of facial fanning and ambient temperature were 2 m/s and 26 °C, respectively, whereas in a previous study, they were 5 m/s and 10 °C, respectively. Additionally, the possibility that T_ty_ was affected by skin temperature could not be completely excluded from the previous study because the experiments did not verify the independence of skin temperature from T_ty_. However, the present study confirmed that T_ty_ was not affected by skin temperature under normothermic conditions. The measurement of T_ty_ in this study also differed from that in the previous study, as the entire ear was covered with sound insulation earmuffs, which could have reduced the effect of skin temperature on T_ty_.

The observed 0.08 °C difference between T_ty_ and T_es_ was very small and fell within the accuracy limit of the thermocouple (±0.1 °C). However, the changes in T_es_ (ΔT_es_) and T_ty_ (ΔT_ty_) from 0 min to 25 min were −0.04 °C and −0.20 °C, respectively, indicating a difference of 0.16 °C between ΔT_es_ and ΔT_ty_ (Table 2), which could not be attributed to measurement error. Moreover, the effect size for the 0.08 °C difference observed at 25 min was large (Cohen's *d* = 2.021; Table 2). Although the 0.08 °C difference was very small, we considered that the difference had physiological significance.

The findings of this study suggest that facial fanning should contribute to brain cooling in individuals with hyperthermia. However, animals with carotid rete can lower their brain temperature by ≥ 1 °C (Hetem et al., 2012; Jessen, 2001; Kuhnen and Mercer, 1993). The brain cooling effect observed in the present study was minimal compared with SBC in animals with carotid rete. SBC in humans could not be validated by the results in this study.

### 4.3 Physiological significance of the preference for low facial temperature in humans

The physiological significance of the preference for low facial temperatures in humans is thought to involve facilitating heat dissipation from the head region. The forehead has one of the highest sweating rates among body regions (Cotter et al., 1995; Kondo et al., 1998; Smith and Havenith, 2012), and these facial characteristics contribute to heat loss from the head. Regarding the regulation of blood flow in the head, skin blood flow increases, whereas central blood flow decreases under hyperthermic conditions (Sato et al., 2011). Blood flow through the emissary and ophthalmic veins moves from the face and scalp into the intracranium, allowing cooled venous blood from the skin to drain toward the cranial cavity (Cabanac and Brinnel, 1985; Hirashita et al., 1992; Nagasaka et al., 1990). Behavioural thermoregulation based on the preference for a low facial temperature—such as voluntarily fanning the face—should enhance heat dissipation through these autonomic thermoregulatory responses and thereby help cool the brain.

### 4.4 Cooling methods and cooling effect

Although the maximum index of brain cooling (difference between T_ty_ and T_es_) was only 0.08 ± 0.04 °C, the results of the present study suggest that facial fanning had a small effect on brain cooling in individuals with hyperthermia. However, brain cooling did not occur in the COND_head_ or COND_neck_ trials. Under normothermic conditions, skin temperature of the cooled site was higher during the FAN_face_ trial than during the COND_head_ and COND_neck_ trials after starting local cooling (Fig. 3). However, under hyperthermic conditions, skin temperature of the cooled site was lower during the FAN_face_ trial than during the COND_head_ and COND_neck_ trials before and after starting local cooling (Fig. 5D). The lower skin temperature at the cooled site during the FAN_face_ trial was elicited by the facilitated evaporation of sweat with facial fanning. In addition, facial fanning can cool the entire head. However, forehead- or neck-conductive cooling with a temperature stimulator (COND_head_ and COND_neck_) inhibits evaporative heat loss from the cooled sites. It can only cool the area to which the stimulator is attached. Hence, forehead or neck-conductive cooling produced a lower cooling effect in this study.

A previous study demonstrated that conductive head cooling for therapeutic hypothermia in patients with acute stroke could achieve SBC (Diprose et al., 2022). In the study, the coolant temperature was maintained at −1.3 °C, and an 80-min cooling period resulted in a 0.9 °C reduction in brain temperature. Although such specialised cooling devices can selectively cool the brain, achieving SBC under natural conditions should be difficult.

### 4.5 Autonomic thermoregulatory responses

If selective brain cooling were to occur, a decline in brain temperature might suppress heat loss responses mediated by hypothalamic control, leading to reduced cutaneous vasodilation and sweating, and potentially resulting in a higher T_es_. Therefore, skin blood flow and sweating were measured in the present study to examine this possibility.

The percentage change in LDF_head_ was lower during the FAN_face_ trial than during the NO-C trial under hyperthermic conditions (Fig. 7B). The lower %LDF_head_ during the FAN_face_ trial may have been elicited by a decrease in the skin temperature of the forehead with facial fanning (Fig. 5D). In contrast, LDF_arm_, %LDF_arm_, and %CVC_arm_ did not differ across the four trials (Fig. 7D–F). With respect to sweating, although ΔSR_arm_ was lower during the FAN_face_ trial than during the COND_neck_ and COND_head_ trials at 5 min (Fig. 8B), T_es_ was also significantly lower during the FAN_face_ trial (Fig. 5A). These results indicate that facial fanning did not facilitate hyperthermia under the conditions tested in this study. The participants wore water-impermeable suits in this study; therefore, differences in sweat rate might not have substantially affected body temperature.

### 4.6 Limitations and future directions

This study involved a small, male-only sample (n = 8). The limited sample size might restrict statistical power; the results cannot be generalized to female individuals. Further studies with a larger sample size and including female participants are required to confirm the present findings.

The present results were obtained under specific experimental conditions. Wearing a tube-lined water-perfused suit and water-impermeable rainwear possibly suppressed natural fluctuations in skin temperature and evaporative heat loss. Under more natural conditions, or with longer cooling durations or lower cooling temperatures, different results might be obtained.

Although we measured T_ty_ as an index of brain temperature in this study, several studies have demonstrated that whole-brain temperature can be measured non-invasively using magnetic resonance thermometry (Rieke and Pauly, 2008). The existence of a thermal gradient within the brain has also been proposed (Mariak et al., 1993, 1999). Further studies measuring whole-brain temperature are necessary to investigate the details of the brain-cooling effect of face or neck cooling.

## 5 Conclusion

This study showed that T_ty_ was not affected by skin temperature and that T_ty_ reflected the precise core body temperature under normothermic conditions. Under hyperthermic conditions, T_ty_ was slightly lower than T_es_ after facial fanning, with the largest difference observed 10 min after fanning; the index of brain cooling (T_es_ – T_ty_) was 0.08 ± 0.04 °C. These results suggest that facial fanning should contribute slightly to brain cooling in hyperthermic individuals. The physiological significance of the preference for low facial temperatures in humans is thought to involve facilitating heat dissipation from the head region and helping cool the brain. However, the effect of brain cooling should be minimal compared to that of SBC in animals with carotid rete. SBC in humans could not be validated by the results of this study.

## Data accessibility statement

Data are available at https://data.mendeley.com/datasets/s2shrhscx6/1.

## CRediT authorship contribution statement

Mayumi Matsuda-Nakamura: Writing – review and editing, Writing – original draft, Visualization, Methodology, Investigation, Funding acquisition, Formal analysis, Data curation. Satoshi Wada: Writing – original draft, Investigation, Formal analysis. Kei Nagashima: Writing – review and editing, Supervision, Resources, Methodology, Funding acquisition.

## Declaration of generative AI and AI-assisted technologies in the writing process

During the preparation of this work, the author used ChatGPT-5 and Claude 3.5 Sonnet for English language editing and enhancement of clarity. After using these tools, the author reviewed and edited the content as needed and took full responsibility for the content of the publication.

## Funding

This work was supported by the 10.13039/501100001691Japan Society for the Promotion of Science, 10.13039/501100001691KAKENHI Grant-in-Aid for Scientific Research A [19H01128], and the 10.13039/501100001691KAKENHI Grant-in-Aid for Young Scientists B [25870831].

## Declaration of competing interest

The authors declare that they have no known competing financial interests or personal relationships that could have appeared to influence the work reported in this paper.
